# Editorial: Emerging talents in pharmacology of anti-cancer drugs 2022

**DOI:** 10.3389/fphar.2023.1191321

**Published:** 2023-04-19

**Authors:** Husain Yar Khan, Khuloud Bajbouj, Mai F. Tolba

**Affiliations:** ^1^ Karmanos Cancer Institute, Wayne State University School of Medicine, Detroit, MI, United States; ^2^ College of Medicine, University of Sharjah, Sharjah, United Arab Emirates; ^3^ Research Institute for Medical and Health Sciences, University of Sharjah, Sharjah, United Arab Emirates; ^4^ CNIO-Spanish National Cancer Research Center, Melchor Fernandez Almagro, Madrid, Spain; ^5^ Department of Pharmacology and Toxicology, Faculty of Pharmacy, Ain Shams University, Cairo, Egypt

**Keywords:** breast cancer, hepatocellular carcinoma, colon cancer, pancreatic cancer, lung cancer

Continuous research efforts have significantly unraveled fundamental molecular pathways that have contributed to paving the way for the development of a multitude of therapeutic strategies, which have and expanded the arsenal of weapons against cancer. This has been tremendously reflected on the uplifting of the cancer survival curve and the improvement of the quality of life of cancer patients. The Research Topic herein was dedicated to highlighting the emerging talents of student researchers pursuing studies in the area of pharmacology of anticancer drugs. The articles in this Research Topic have shed light on the quality and diversity of student researchers across this field and focused on three main themes: 1) Remodeling Cancer Epigenome: Therapeutic Advances; 2) Cancer Immunosurveillance: From Mechanisms to Therapy; 3) Anticancer Drug Discovery and Translational Oncology. The studies herein include two reports of clinical trials by Tan et al. and Zhu et al. A diversity of preclinical/translational research studies were also presented in the Research Topic. Lozon et al. presented a novel chemotherapy combination that was validated *in vitro* in colon cancer and lung cancer cell lines. Abdollah et al., Yang et al., and Lee et al. highlighted a promising anticancer activity for a variety of natural products against hepatocellular carcinoma. On the other hand, the work of Lee et al., Lin et al., Irshad et al., and Kuttikrishnan et al. presented mechanistic insights in pancreatic cancer, breast cancer, non-small-cell lung cancer, and leukemia. The Research Topic also encompasses two literature reviews by Bannoura et al. and Yuan et al.

Trastuzumab deruxtecan (T-DXd), an antibody–drug conjugate, has shown antitumor activity in patients with HER2-low advanced breast cancer (ABC) in clinical trials. However, the cost-effectiveness of T-DXd was not clearly established. In order to determine whether the benefits of this treatment outweigh its costs, Zhu et al. employed a Markov decision-analytic model to compare the cost-effectiveness of T-DXd with chemotherapy in HER2-low ABC patients. While T-DXd showed improved quality-adjusted life years (QALYs), its incorporation led to increased costs and an unfavorable incremental cost-effectiveness ratio (ICER) compared to chemotherapy, making it devoid of cost-effectiveness in HER2-low ABC patients in the United States. The authors, however, believe that it may still provide health benefits to patients with HR+/HER2-low ABC.

In their case report, Tan et al. suggested that pembrolizumab plus nab-paclitaxel might be a potential treatment option for patients with cholangiocarcinoma (CCA), which is a highly aggressive malignancy with poor overall survival. In this report, a metastatic extrahepatic CCA patient achieved durable response and good tolerance to a combination treatment of pembrolizumab plus nab-paclitaxel following progression on a chemotherapy of gemcitabine and capecitabine.

Lozon et al. contributed an original article highlighting the importance of safranal (SAF) as a promising candidate-sensitizing agent of human colon cancer cells (HCT116) and lung cancer cells (A549) to the cytotoxic effect of the topoisomerase inhibitor topotecan (TPT). The combination augmented TPT-induced alterations in DNA repair and boosted the incidence of double-strand breaks with subsequent induction of apoptosis. The sensitization only occurred when the cells were pretreated with SAF 24 h before TPT treatment. However, simultaneous exposure or adding SAF 24 h after TPT did not recapitulate the same results. Hence, the effect was sequence dependent.

An article by Abdollah et al. aimed to determine whether fucoidan’s chemomodulatory benefits may be enhanced by using it in combination with the FDA-approved antiangiogenic drugs sorafenib and Avastin (bevacizumab) in order to augment their anticancer activity in hepatocellular carcinoma (HCC) cells. The authors reported that the combination treatment inhibited the PI3K/AKT/mTOR and KRAS/BRAF/MAPK pathways in addition to apoptosis induction in HCC both *in vitro and in vivo*.

Yang et al. identified a potential pharmacological mechanism by which Ganji Fang (GJF) can treat HCC at the systemic level. The underlying mechanisms were shown to involve immune control, cell migration, cell proliferation, apoptosis, and inflammation induction. Furthermore, it was demonstrated that the G0/G1 phase cycle arrest subsequent to the apoptosis after GJF treatment in liver cancer cells was triggered by blocking the PI3K/Akt signaling pathway. Pachymic acid has been shown to be the significant active component of GJF that exhibits anticancer action, and EPHA2 may be a potential key target for GJF in HCC.

It was previously found that white genius mushroom (WGM), a popular edible mushroom in Taiwan, mediates strong antiproliferative activities against human Hep3B liver cancer cells. However, the underlying mechanisms have not been fully investigated. Lee et al. reported that WGM extracts induced cell death by targeting mTOR and MAPK signaling pathways in liver cancer cells, suggesting that they could be used as a pharmacologically safe natural dietary chemopreventive agent for HCC treatment.

The article by Lee et al. investigated the efficacy and molecular mechanisms of ivermectin/gemcitabine combination in pancreatic cancer. They observed that a combination treatment of ivermectin and gemcitabine suppressed pancreatic cancer both *in vitro* and *in vivo* more effectively than gemcitabine alone. Mechanistically, this combination was found to exert its effects by inhibiting cell proliferation *via* G1 cell cycle arrest and augmenting apoptosis by inducing mitochondrial dysfunction. Based on these findings, the study concluded that ivermectin, an antiparasitic drug, can exhibit synergistic effects with gemcitabine and may be repurposed to serve as a promising therapeutic agent for pancreatic cancer therapy.

Lin et al. demonstrated the anticancer effects of gallic acid, a phenolic acid known for its antioxidant properties, against triple-negative breast cancer cells *in vitro*. The study showed that gallic acid was able to inhibit the growth of HCC1806 cells and stimulate their apoptosis by triggering the production of reactive oxygen species, which modulate the PI3K/AKT/EGFR and MAPK signaling pathways.

Irshad et al. employed integrative network pharmacology, molecular docking, and *in vitro* experiments to elucidate the mechanism of action of glycyrrhetinic acid (18α-GA), a triterpenoid found in licorice against non-small-cell lung cancer (NSCLC). Their network pharmacology study identified EGFR, AKT1, PI3KR1, MAPK1, IGF1, and SRC as crucial hub targets for 18α-GA against NSCLC. The authors further showed that 18α-GA augmented G1 cell cycle arrest, triggered apoptosis, reduced the migratory potential, and inhibited the EGFR-PI3K/AKT pathway in NSCLC cell lines.

Kuttikrishnan et al. explored the role of neosetophomone-B (NSP-B), a meroterpenoid fungal secondary metabolite, on the FOXM1/BUB1B signaling pathway. The development and progression of various types of cancer, including chronic myelogenous leukemia, have been linked to the abnormal expression of FOXM1 and BUB1B genes. A TCGA data analysis elaborated that BUB1B is overexpressed in most cancers and linked with poor prognosis. Using gene expression profiling, the authors showed the significant downregulation of BUB1B in leukemia cells treated with NSP-B. In addition, they validated their *in silico* findings *in vitro* by showing that NSP-B suppresses the expression of FOXM1 and BUB1B in a dose-dependent manner, leading to compromised cell viability and apoptosis induction in leukemia cells.

The minireview by Bannoura et al. summarized the recent data on targeting KRAS G12D, which is among the most common mutations (45%) in pancreatic cancer that are associated with poor prognosis. The article discussed several modalities under development for targeting KRAS G12D, including small molecule inhibitors and immunotherapy.

Yuan et al. contributed a bibliometric article of the literature from the Web of Science Core Research Topic (2002–2021) on the emerging trends and research foci of the natural compound berberine in cancer. Berberine is a multitarget Chinese medicine monomer compound that is extensively studied for its antitumor/antiproliferative effects and its capacity to sensitize cancer cells to chemotherapy. The collected data showed that berberine exhibits anticancer capacity through a diversity of mechanisms, including halting the cell cycle, inhibition of tumor cell invasion and migration, and inducing autophagy and apoptotic cell death.

